# Time series analysis of in vivo cardiac MRI-PET image fusion of the human amniotic mesenchymal stem cell (hAMSC) engraftment

**DOI:** 10.1186/1532-429X-16-S1-P358

**Published:** 2014-01-16

**Authors:** Karl T Diedrich, Yuka Matsuura, Nicholas Herlambang, Rajesh Dash, Phillip Yang

**Affiliations:** 1 AZE Technology, Inc., Cambridge, Massachusetts, USA; 2 Division of Cardiovascular Medicine, Stanford University School of Medicine, Stanford, California, USA

## Background

This study developed a system to monitor intensity changes in the cardiac PET-MRI images of transplanted stem cells in vivo. A fusion system, consisting of 2 medical imaging modalities to monitor the changes in signal intensity in regions of interest (ROI) over time, was achieved.

## Methods

Porcine ischemia reperfusion (IR) injury model was employed to assess the successful stem cell engraftment signal by manganese enhanced MRI (MEMRI). To validate the MEMRI signal, hAMSCs were transduced by PET reporter gene (RG) utilizing the herpes simplex virus-thymidine kinase transgene construct, which is expressed only in engrafted cells to trap 18F-FHBG radiotracer. One week after IR injury, the RG transduced hAMSCs were delivered via transendocardial injection into peri- and intra-infarct regions. PET-CT and MR locator-MEMRI-delayed enhanced MRI (DEMRI) images were acquired post hAMSC injection on days 0, 7, and 45. All pairs of CT and MR locator images were registered by gradient descent to optimize mutual information between the images generating transformation matrixes. These transformation matrixes were used to align PET with MEMRI and DEMRI and longitudinal images into common frames of reference. Additionally image pairs were fused into a single display. An ROI around the engrafted stem cells on MEMRI was copied to the same location on PET-CT longitudinally and average intensity was sampled at each time point.

## Results

**Figure 1:**
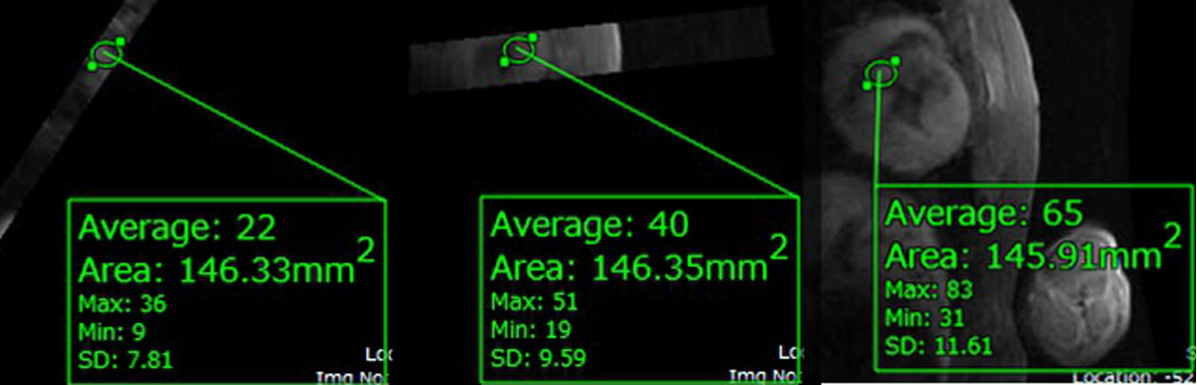
**Average non-normalized signal intensity over an ROI was measured across post stem cell engraftment day 0 (left), 7, (center), 45 (right)**.

A fused PET-MEMRI and PET-DEMRI image, which displayed the two signals in a single image, was generated. The mean normalized ROI intensities across days 0, 7, and 45 were 22.00, 59.37, and 64.37 for MEMRI (Figure 1) and 3263.00, 7733.719, and 3664.310 for PET.

## Conclusions

**Figure 2:**
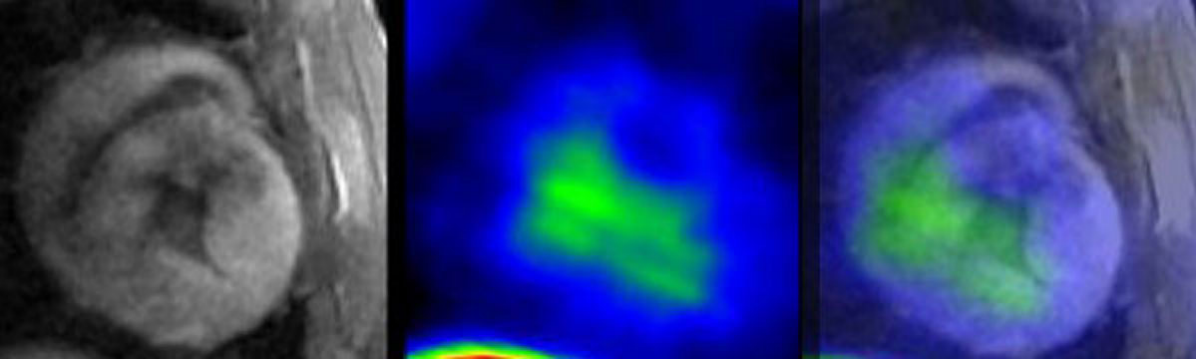
**MEMRI (left) and PET (center) were fused (right)**.

The engrafted hAMSCs demonstrate significant manganese and radiotracer uptake to generate their respective signals indicative of cell engraftment. The increased MEMRI signal volume over time represented cell proliferation. The in vivo PET-MRI fusion validated the visual correlation of cell engraftment MEMRI signal and of longitudinal cell proliferation (Figure 2). This study developed a system that monitors the engraftment of the transplanted stem cells in vivo, employing 2 imaging modalities. The fused PET-MRI images enable high spatial and temporal resolution of MRI while maintaining the high signal sensitivity of PET, demonstrating successful engraftment and proliferation of the stem cells.

## Funding

AZE Technology, Inc. Stanford University.

